# Mutual optical intensity propagation through non-ideal two-dimensional mirrors

**DOI:** 10.1107/S1600577523006343

**Published:** 2023-08-23

**Authors:** Xiangyu Meng, Yong Wang, Xianbo Shi, Junchao Ren, Weihong Sun, Jiefeng Cao, Junqin Li, Renzhong Tai

**Affiliations:** a Shanghai Advanced Research Institute, Chinese Academy of Sciences, 239 Zhangheng Road, Pudong District, Shanghai 201800, People’s Republic of China; b Shanghai Institute of Applied Physics, Chinese Academy of Sciences, 239 Zhangheng Road, Pudong District, Shanghai 201800, People’s Republic of China; c Advanced Photon Source, Argonne National Laboratory, 9700 South Cass Avenue, Argonne, IL 60439, USA; Tohoku University, Japan

**Keywords:** synchrotron beamline, partially coherent light, mutual optical intensity, two-dimensional mirrors

## Abstract

The mutual optical intensity (MOI) model is a partially coherent radiation propagation tool that can sequentially simulate beamline optics and provide beam intensity, local degree of coherence and phase distribution at any location along a beamline. This paper extends the MOI model to non-ideal two-dimensional (2D) optical systems, such as ellipsoidal and toroidal mirrors with 2D figure errors. Simulation results show that one can tune the trade-off between calculation efficiency and accuracy by varying the number of wavefront elements. The focal spot size of an ellipsoidal mirror calculated with 100 × 100 elements gives less than 0.4% deviation from that with 250 × 250 elements, and the computation speed is nearly two orders of magnitude faster. Effects of figure errors on 2D focusing are also demonstrated for a non-ideal ellipsoidal mirror and by comparing the toroidal and ellipsoidal mirrors. Finally, the MOI model is benchmarked against the multi-electron *Synchrotron Radiation Workshop* (*SRW*) code showing the model’s high accuracy.

## Abstract 2

Mutual optical intensity propagation through non-ideal two-dimensional mirrors is realized by combining geometric ray-tracing and wavefront propagation.

## 1. Introduction

Free-electron lasers (Barty *et al.*, 2009 ▸) and diffraction-limited storage rings (Eriksson *et al.*, 2014 ▸) have been developed worldwide, with high photon brightness and high coherence properties. Accurate simulation of partially coherent X-ray propagation through a beamline can help determine the optimal detector location and estimate the X-ray intensity distribution on the detector (Chubar *et al.*, 2011 ▸; Canestrari *et al.*, 2014 ▸). Also, experiments (Whitehead *et al.*, 2009 ▸; Meng *et al.*, 2021 ▸) exploiting radiation coherence need to obtain the degree of coherence and flux at the sample location to develop experimental data processing algorithms and accurately interpret experimental results.

Several software packages have been developed to calculate partially coherent radiation propagation through beamlines, such as *Synchrotron Radiation Workshop* (*SRW*) (Samoylova *et al.*, 2011 ▸; Chubar, 2014 ▸) and *XRT* (Khubbutdinov *et al.*, 2019 ▸) using the multi-electron approach, *Comsyl* (Glass & Sanchez del Rio, 2017 ▸) based on coherence mode decomposition, and *HYBRID* (Shi *et al.*, 2014 ▸) combining ray-tracing and wavefront propagation. Recently, we developed a new model (Meng *et al.*, 2015 ▸, 2017 ▸), which uses the mutual optical intensity (MOI) to describe the partially coherent X-ray radiation following the statistical optics theory. This MOI model propagates the MOI function through beamline optics and provides the beam intensity, coherence and phase information at a specified beamline position. Unlike the multi-electron methods, the MOI model has the advantage of storing the full MOI functions at any beamline location for sequential simulation and interpretation of results.

We have already shown that the MOI model can simulate non-ideal mirrors with one-dimensional (1D) figure error profiles (Meng *et al.*, 2017 ▸), mainly working for cylindrical mirrors. This paper extends the MOI model to deal with non-ideal two-dimensional (2D) optical systems, such as ellipsoidal mirrors, toroidal mirrors and Kirkpatrick–Baez (KB) mirror pairs. We use the MOI model to demonstrate the partially coherent light propagation through ellipsoidal and toroidal mirrors by studying beam intensity, degree of coherence and phase distribution at the focal plane with and without mirror figure errors. We also show that the calculation accuracy could be improved by increasing the number of wavefront elements at the cost of simulation time. Since the full coherence function is stored, the fast rough estimation and slow accuracy calculation can be switched as needed, making the MOI model a powerful beamline design tool. Finally, the MOI model is benchmarked against *SRW* in simulating the focusing property of a toroidal mirror.

## 2. Model description

### 2.1. MOI propagation through free space

The partially coherent beam is described by the MOI, *J*(*P*_1_, *P*_2_), which is a four-dimensional function describing the electric field distribution and the correlation between any two points *P*_1_ and *P*_2_. The MOI propagation through free space can be represented by the equation (Mandel & Wolf, 1995 ▸, Goodman, 2015 ▸)

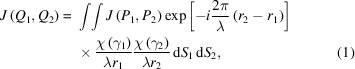

where *J*(*P*_1_, *P*_2_) and *J*(*Q*_1_, *Q*_2_) represent the MOI at the object and image planes, respectively, λ is the wavelength, *S*_1_ and *S*_2_ denote the surface of the object plane, *Q*_1_ and *Q*_2_ are any two points at the image plane, *r*_1_ and *r*_2_ are the *P*_1_-to-*Q*_1_ and *P*_2_-to-*Q*_2_ distances, respectively, and χ(γ_1_) and χ(γ_2_) are the inclination factors for the inclination angles γ_1_ and γ_2_, respectively. The intensity can be extracted from the MOI and can be expressed as

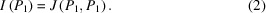

In the MOI model, the wavefront is separated into many small elements to quantitatively solve the MOI propagation through free space. When the element size is much smaller than the beam size and the transverse coherence length, the beam within a single element can be reasonably considered to have full coherence and constant intensity. However, the beam between any two elements is partial coherent. Therefore, the propagation of each element of the MOI can be carried out using the Fraunhofer or Fresnel approximation (Born & Wolf, 1999 ▸). Finally, the MOI at the image plane is obtained by numerically summing the contributions of all elements.

The source coherence lengths ξ can be calculated from (Vartanyants & Singer, 2010 ▸)

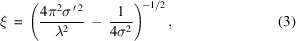

where σ′ and σ are the r.m.s. divergence angle and r.m.s. size, respectively. The normalized global degree of coherence of the 2D source can be obtained by using (Vartanyants & Singer, 2010 ▸)

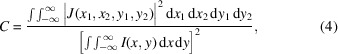

where *I*(*x*, *y*) is the intensity distribution of the 2D source. The known coherence length ξ and source r.m.s. size σ mean that the degree of coherence and intensity are both Gaussian distributions. Therefore, equation (3) is valid only for a Gaussian Schell model source. The MOI *J*(*x*_1_,*x*_2_,*y*_1_,*y*_2_) is independent of source type and can describe the degree of coherence distribution more generally. Therefore, the global degree of coherence can more accurately define the coherence property.

### 2.2. MOI propagation through mirrors

**Figure 1:**
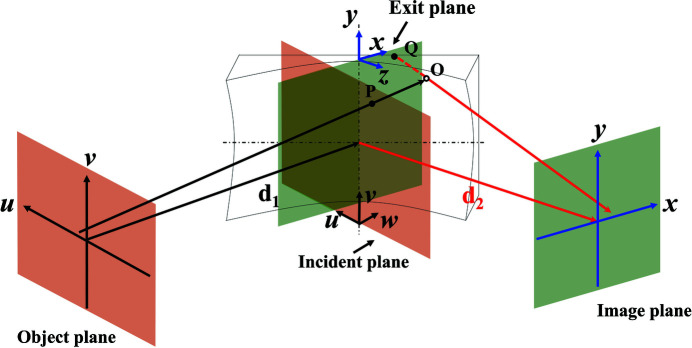
Schematic layout of the MOI propagation through a 2D mirror.

The MOI model uses local ray tracing (Meng *et al.*, 2017 ▸) to analyze the path length distribution caused by the mirror surface. The path length is the travel distance of a ray from the incident plane to the exit plane. The incident and exit planes are at zero distance from the mirror center and perpendicular to the incident and exit optical axes, respectively, as shown in Fig. 1 ▸. The direction of a ray is defined by the local phase gradient of the wavefront. The MOI model evaluates the path length distribution of all rays that impinge on and reflect off the 2D mirror surface. The spatial coordinates of the incident and exit planes are defined as *P*(*u*, *v*, *w*) and *Q*(*x*, *y*, *z*), respectively. The transverse axes *u*, *x* and *v*, *y* define the horizontal and vertical directions, respectively, while the longitudinal axes *w* and *z* define beam propagation directions. *d*_1_ and *d*_2_ are the object-to-mirror and the mirror-to-image distances, respectively. Using *J*_i_(*Q*_1_, *Q*_2_) and *J*_e_(*Q*_1_, *Q*_2_) to represent the MOI at the mirror incident and exit planes, respectively, the MOI propagation through a 2D mirror can be expressed as

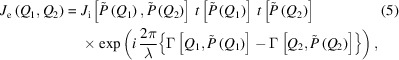

where Γ(*Q*, *P*) is the path length between the incident and exit planes. 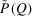 is the coordinate transformation function mapping the exit plane point to the incident plane through ray tracing. Since this coordinate transformation is non-linear on non-regular grids, the amplitude of the wavefront needs to be scaled accordingly through the complex amplitude transmission function 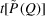 given by

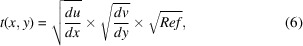

where *Ref* is the intensity reflectivity based on the incident angle, which is assumed to be 1 in this case due to the small incident angle. *du* and *dv* are local coordinate step sizes at the incident plane; *dx* and *dy* are local coordinate step sizes at the exit plane. 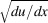 and 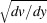 are used to scale the amplitude of the wavefront from the incident plane element to the corresponding exit plane element. Finally, the MOI propagation from the object plane to the image plane can be carried out by a three-step sequential propagation, including an object-to-incident plane free-space propagation using equation (7) in Appendix *A* [App appa], an incident-to-exit plane mirror propagation based on equation (5), and an exit-to-image plane free-space propagation using equation (7).

## 3. Partially coherent light propagation through 2D mirrors

### 3.1. Mutual optical intensity propagation through an ellipsoidal mirror

The BL08U1B beamline at the Shanghai Synchrotron Radiation Facility (SSRF) generates high coherent X-rays for the soft X-ray interference lithography (XIL) experiment (Xue *et al.*, 2018 ▸). The beamline uses two cylindrical mirrors to focus the beam at the exit slit plane at 26 m. An elliptically polarized undulator (EPU) with a length of 4.2 m and a period of 100 mm is used to generate the high-brilliance and partially coherent beam.

The MOI of the source is described by the Gaussian Schell model (Vartanyants & Singer, 2010 ▸). At 92.5 eV, the photon source has r.m.s. sizes of σ_*x*_ = 155 µm and σ_*y*_ = 54.4 µm and r.m.s. divergence angles of 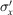 = 51.7 µrad and 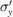 = 40.2 µrad, calculated using *SPECTRA* (Tanaka & Kitamura, 2001 ▸), with *x* and *y* denoting the horizontal and vertical directions, respectively. The source coherence lengths are ξ_*x*_ = 41.6 µm and ξ_*y*_ = 60.8 µm, calculated by using equation (3).

The normalized global degree of coherence of the 2D source is obtained to be *C* = 0.067 by using equation (4). In this section, a horizontally deflecting ellipsoidal mirror is designed to replace the two cylinder mirrors at 22 m downstream of the EPU source. The length and width of the ellipsoidal mirror are 88 mm and 1.8 mm, respectively, with a grazing-incident angle of 1.5°. The source (object)-to-mirror and mirror-to-focus (image) distances are *d*_1_ = 22 m and *d*_2_ = 4 m, respectively. The semi-major axis, the semi-minor axis and the pole angle of the ellipsoid mirror are 13 m, 0.2456 m and 1.1275°, respectively. The beam is focused horizontally (in the meridional direction of the mirror) and vertically (in the sagittal direction of the mirror) into the exit slit location (image plane) at 26 m from the source.

**Figure 2:**
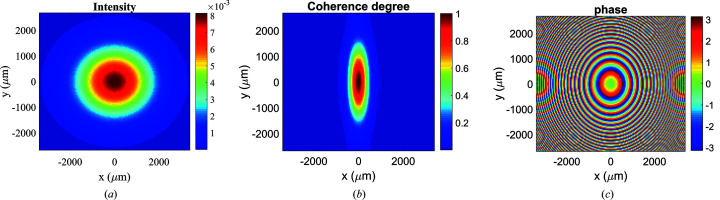
(*a*) Intensity, (*b*) degree of coherence between any point and the central point and (*c*) phase distributions shift relative to the center point at the mirror incident plane.

The MOI propagation through the ellipsoidal mirror is carried out using the MOI model, with a wavefront element number of 150 × 150. The extracted beam intensity, local degree of coherence and phase distributions at the mirror incident plane are shown in Fig. 2 ▸. The beam intensity profile [Fig. 2 ▸(*a*)] at the incident plane is approximately a Gaussian distribution with r.m.s. sizes of σ_*x*_ = 1147.3 µm and σ_*y*_ = 893.9 µm. The local degree of coherence distribution between any points and the central point is approximately a Gaussian distribution as well, as shown in Fig. 2 ▸(*b*). The coherence lengths are ξ_*x*_ = 308.5 µm and ξ_*y*_ = 993.2 µm obtained by Gaussian fitting of the degree of coherence distribution relative to the center point on the *x* and *y* axes, respectively. The global degree of coherence obtained from equation (4) remains the same value (0.067) as the source since the free-space propagation does not alter the beam coherence. Note that the MOI model can also give the local degree of coherence value between any two points. The phase distribution [Fig. 2 ▸(*c*)] at the mirror incident plane is a divergent spherical wave with a spherical wave radius of 22 m. The phase distribution is shifted relative to the center point. The concentric feature is from the phase wrapping.

**Figure 3:**
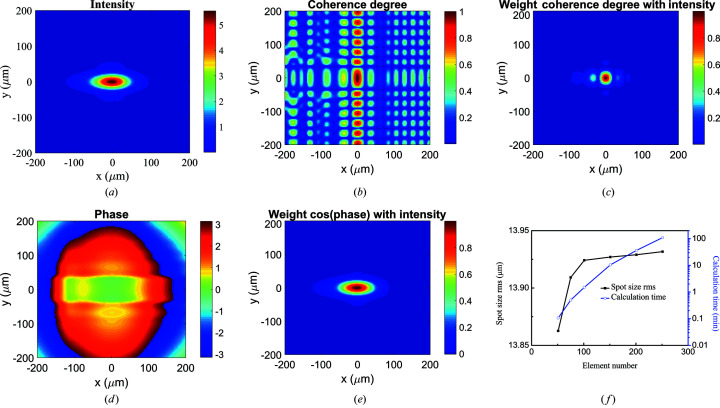
(*a*) Intensity, (*b*) degree of coherence between any point and the central point, (*c*) intensity-weighted degree of coherence distribution, (*d*) phase distributions at the focal plane and (*e*) intensity-weighted cos(phase). (*f*) Intensity spot size σ_*y*_ (black square line) in the vertical direction at the image plane and calculation time (blue circle line) as a function of the wavefront element number (in each *x* and *y* direction) used in the MOI model.

From the MOI at the focal plane, the intensity, degree of coherence and phase distributions are extracted and shown in Figs. 3 ▸(*a*)–3(*e*). The intensity profile [Fig. 3 ▸(*a*)] at the focal plane is still Gaussian with an r.m.s. spot size of σ_*x*_ = 33.9 µm and σ_*y*_ = 13.9 µm. Due to the diffraction effect from the finite size of the ellipsoidal mirror, one can find apparent oscillations in the degree of coherence image in Fig. 3 ▸(*b*). For simplicity and relative comparison only, we define the coherence length as FWHM/2.35 of the central peak of the degree of coherence distribution and extract the global degree of coherence value using equation (4). Then the coherence lengths at the focal plane are ξ_*x*_ = 10.7 µm and ξ_*y*_ = 23.5 µm, and the corresponding global degree of coherence is 0.17. This increase in the global degree of coherence compared with the value (0.067) at the mirror incident plane is a result of the finite aperture of the mirror acceptance. The degree of coherence has high value in the edge regions where the intensity is almost zero, as shown in Fig. 3 ▸(*b*). The regions with high coherence but low intensity have no interest to us. Therefore, the degree of coherence weighted by intensity is used to describe the available degree of coherence distribution. The degree of coherence distribution with the intensity-weighted transparency in Fig. 3 ▸(*c*) shows a smaller central region in the *y*-axis direction compared with Fig. 3 ▸(*b*), indicating that the beam is nearly fully coherent in the vertical direction. The phase distribution in Fig. 3 ▸(*d*) shows the cosine of the phase shift relative to the center point. We define the flat plane wave size as where the cos(phase) value is larger than 0.98 for comparison only. The flat plane wave size is 81.1 µm (H) × 107.7 µm (V), indicating the excellent point-to-point focusing property of the ideal ellipsoidal mirror. Like the degree of coherence distribution with intensity-weighted transparency, the region with low intensity has no interest to us, so we added the cos[φ(*Q*_1_)] weighted with intensity where φ(*Q*_1_) is the phase at the point *Q*_1_. Due to the flat plane wave size being larger than the spot size, the cos(phase) distribution with the intensity weighted transparency shown in Fig. 3 ▸(*e*) has a similar profile to the intensity distribution [Fig. 3 ▸(*a*)].

Since the efficiency and accuracy of the numerical simulation are crucial for the beamline design, we use the above case to study the effects of wavefront element numbers. The intensity spot size (black square line) in the vertical direction at the image plane and simulation time (blue circle line) from source to focus are plotted as a function of the element number in Fig. 3 ▸(*f*). Increasing the element number improves the simulation accuracy at a cost of calculation time. The calculation with 100 × 100 elements gives less than 0.4% deviation from the saturation value obtained with 250 × 250 elements, which is acceptable for most beamline design cases. The computation time is 1.5 min with 100 × 100 elements compared with 1.8 h with 250 × 250 elements. More importantly, one can choose the balance between the accuracy and efficiency of the MOI model by changing the element number at each calculation plane, which is very user-friendly for beamline designers. All MOI simulations shown here were performed on a single NVIDIA A100 Tesla GPU (80 GB RAM). The calculation efficiency can be further improved by adding more GPUs or algorithm optimization.

### 3.2. MOI propagation through non-ideal 2D mirrors

**Figure 4:**
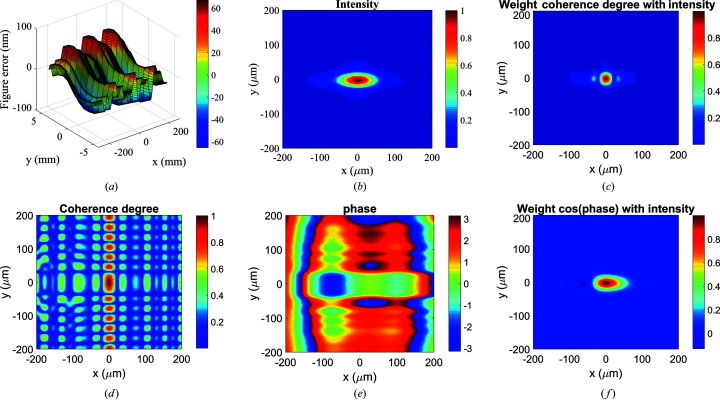
(*a*) Figure error of the ellipsoidal mirror, (*b*) intensity, (*c*) degree of coherence between any point and the central point, (*d*) intensity-weighted degree of coherence distribution, (*e*) phase distributions at the focal plane and (*f*) intensity-weighted cos(phase).

Using geometric ray tracing to directly calculate the path length for the mirror with figure error, the MOI model can analyze the MOI propagation through a non-ideal mirror as shown for a 1D system (Meng *et al.*, 2017 ▸). Here, we extend the MOI model and demonstrate the model capability by simulating a non-ideal 2D mirror. The 2D figure error profile with an r.m.s. height error of 20 nm was generated using the height profile simulator module in *OASYS* (Rebuffi & Sanchez del Rio, 2017 ▸) and shown in Fig. 4 ▸(*a*). The corresponding slope errors in the *x* (meridional) and *y* (sagittal) directions are 2 µrad and 10 µrad, respectively.

The MOI propagation was then carried out with the figure error added to the ellipsoidal mirror in Section 3.1. The extracted intensity distribution at the focal plane is shown in Fig. 4 ▸(*b*). The r.m.s. spot sizes are σ_*x*_ = 35.6 µm and σ_*y*_ = 14.0 µm. Compared with the spot sizes of σ_*x*_ = 33.9 µm and σ_*y*_ = 13.9 µm for the ideal mirror case in Fig. 3 ▸(*a*), the figure error has a larger effect on the beam focus in the mirror meridional direction, as expected for the grazing incident optics. Fig. 4 ▸(*c*) shows the degree of coherence between any point and the central point. The coherence lengths at the focal plane are ξ_*x*_ = 11.6 µm and ξ_*y*_ = 23.9 µm, and the corresponding global degree of coherence is still 0.17. Thus the figure error can change the degree of coherence distribution at the focal plane but not the global degree of coherence. The degree of coherence distribution with the intensity-weighted transparency shown in Fig. 4 ▸(*d*) again shows a smaller central region in the *y* direction. Fig. 4 ▸(*e*) shows the phase distribution at the focal plane. The plane wave part has a size of 61.5 µm (H) × 71.5 µm (V), noticeably smaller than that in Fig. 3 ▸(*d*) for the ideal mirror, which indicates that the figure error can distort the wavefront and reduce the plane wave size. This knowledge of local coherence and phase distribution can be helpful for beamline experiment design and optimization. The cos(phase) distribution with the intensity-weighted transparency is shown in Fig. 4 ▸(*f*), which has apparent asymmetry in the *x* direction resulting from the figure error effects.

### 3.3. Focusing properties of ellipsoidal and toroidal mirrors

**Figure 5:**
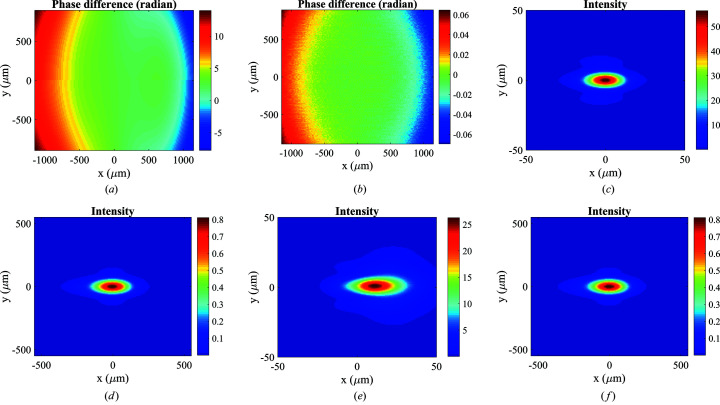
Phase difference between the ellipsoidal and toroidal mirrors for two mirror-to-image distances (*a*) *d*_2_ = 1 m and (*b*) *d*_2_ = 11 m. Intensity distribution of the focal spot for the ellipsoidal mirror for two mirror-to-image distances *d*_2_ = 1 m (*c*) and *d*_2_ = 11 m (*d*). Intensity distribution of the focal spot for the toroidal mirror for two mirror-to-image distances (*e*) *d*_2_ = 1 m and (*f*) *d*_2_ = 11 m.

This section compares intensity distributions at the focal plane of an ellipsoidal mirror and a toroidal mirror using the MOI model with two mirror-to-image distances, *d*_2_ = 1 m and 11 m. A layout sketch describing the beam propagation through the ellipsoidal or toroidal mirrors is shown in Fig. 1 ▸. For the toroidal mirror with *d*_2_ = 11 m, the major and minor radii are 560.2894 m and 0.3839 m, respectively. For the toroidal mirror with *d*_2_ = 1 m, the major and minor radii are 73.0812 m and 0.050 m, respectively. The induced phase difference between the ellipsoidal and toroidal mirrors is given by φ = 2π/λ(Γ_ell_ − Γ_tor_), where Γ_ell_ and Γ_tor_ are the path lengths between the incident and exit planes for the ellipsoidal and toroidal mirrors, respectively. Figures 5 ▸(*a*) and 5 ▸(*b*) show the phase difference between the two mirrors for *d*_2_ = 1 m and 11 m, respectively. The ellipsoidal mirror is considered the ideal shape for point-to-point focusing with any magnification factor. On the other hand, the toroidal mirror can only provide perfect focusing with a 1:1 magnification, or *d*_2_ = *d*_1_. The larger the difference between *d*_1_ and *d*_2_, the more prominent aberration (deviation from the ideal ellipsoidal shape) the toroidal mirror suffers from. Thus the phase difference for the *d*_2_ = 1 m case in Fig. 5 ▸(*a*) is much bigger than the *d*_2_ = 11 m case in Fig. 5 ▸(*b*). Figures 5 ▸(*c*) and 5 ▸(*d*) show the intensity distribution at the focal plane of the ellipsoidal mirror for the mirror-to-image distances *d*_2_ = 1 m and 11 m, respectively, while Figs. 5 ▸(*e*) and 5 ▸(*f*) show the results of the toroidal mirror. For the *d*_2_ = 1 m case, the r.m.s. spot size is 8.4 µm (H) × 3.6 µm (V) for the ellipsoidal mirror [Fig. 5 ▸(*c*)], compared with 12.5 µm (H) × 4.3 µm (V) for the toroidal mirror [Fig. 5 ▸(*e*)]. The focal spot of the toroidal mirror shows apparent coma aberration in the meridional (horizontal) direction. The broadening effect in the sagittal direction is less but still notable. Thus, the capability of simulating in 2D is essential for these 2D focusing mirrors, especially for evaluating the effect of 2D figure errors. For the *d*_2_ = 11 m case, both mirrors provide a similar spot size of 92.3 µm (H) × 38.2 µm (V) [*cf*. Figs. 5 ▸(*d*) and 5 ▸(*f*)]. These simulation results are consistent with the geometric-optics description. Furthermore, the MOI propagation captures all the wave-optics phenomena, such as predicting a larger spot size than the demagnified source size by including the diffraction effect of the finite mirror acceptance aperture.

### 3.4. Benchmarking the MOI model against *SRW*

**Figure 6:**
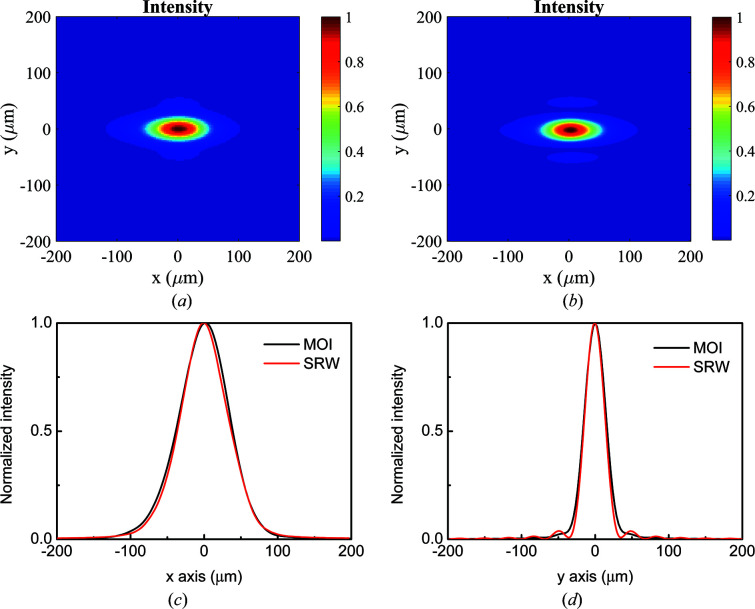
Intensity distribution at the focal plane of a toroidal mirror obtained from (*a*) the MOI model and (*b*) the *SRW* simulation. Integrated intensity profiles in (*c*) the horizontal direction and (*d*) the vertical direction using the MOI model (black curves) and *SRW* (red curves).

We use the toroidal mirror as an example to benchmark the MOI model against multi-electron *SRW* simulation. The toroidal mirror has a grazing-incident angle of 1.5° with object distance of 22 m and image distance of 4 m. In *SRW*, the partial coherence of the beam is taken into account by propagating the radiation from individual micro-electrons and sums up intensities at a given observation plane (Chubar, 2014 ▸). In the MOI case, 150 × 150 wavefront elements were considered. In the *SRW* case, 5000 electrons were considered. The comparison results of the two methods are shown in Fig. 6 ▸. The intensity distribution at the focal plane extracted from the MOI model in Fig. 6 ▸(*a*) has an r.m.s. spot size of 33.8 µm (H) × 14.0 µm (V), while the focal spot size from *SRW* calculation in Fig. 6 ▸(*b*) is 32.6 µm (H) × 12.6 µm (V). Figures 6 ▸(*c*) and 6 ▸(*d*) compare the integrated intensity profiles from the two methods in the horizontal and vertical directions, respectively. The MOI model and *SRW* calculation generally give intensity profiles in close agreement. The slight difference between their results is insignificant for most beamline design and simulation applications. The deviation is acceptable considering that the two models use very different approaches in simulating the undulator source, propagating through free space and optics, and extracting results. The simulation times for the MOI model and the multi-electron *SRW* are 10.4 min and 172 min, respectively.

## 4. Conclusions

As an advanced mutual coherence simulation tool, the MOI model aims to provide complete information on the partially coherent radiation, including intensity, degree of coherence and wavefront phase, with high accuracy and reasonable computation efficiency. Modern computer development makes it possible to numerically integrate the four-dimensional MOI. Since the MOI is stored at any calculated plane, beamline simulation can be conducted stepwise with optimized parameters for each step. Changing the number of wavefront elements allows switching between fast estimation and accurate simulation, making the MOI model a user-friendly beamline design tool.

In this paper, the MOI model is extended to include non-ideal two-dimensional optics, where the robustness and efficiency are even more critical due to the large data volume. It is thus essential to show that simulation with 100 × 100 elements can provide sufficiently accurate results (<0.4% deviation from the saturation value with 250 × 250 elements) with nearly two orders of magnitude higher speed. Using the MOI model, we simulated the beam propagation through ellipsoidal and toroidal mirrors with and without figure errors. The MOI model can successfully describe the effects of 2D mirror figures and height errors by calculating the path length through local ray tracing. Finally, the MOI model is benchmarked against *SRW* with good agreement. With the extension to 2D mirrors, the MOI model covers an extensive range of advanced beamline optics and is a powerful tool for coherence-related beamline simulation.

## Appendix A

The MOI at the image plane is obtained by numerically summing the contributions of all elements and can be expressed as

where *j*, *k*, *m* and *n* are the element indexes at the object plane, and with

where φ(*S*_*jk*_) is the phase distribution within the element surface *S*_*jk*_.

The MOI can be expressed as

where *I*(*P*_1_) and *I*(*P*_2_) are the intensities at the points *P*_1_ and *P*_2_, respectively, |μ_12_(*P*_1_, *P*_2_)| is the degree of coherence between the two points *P*_1_ and *P*_2_, and φ_12_ is the phase difference between the two points. One can extract the intensity, local degree of coherence and phase distribution from the MOI.
